# Comparison of *Enterococcus faecalis* Biofilm Removal Efficiency among Bacteriophage PBEF129, Its Endolysin, and Cefotaxime

**DOI:** 10.3390/v13030426

**Published:** 2021-03-07

**Authors:** Hyun Keun Oh, Yoon Jung Hwang, Hye Won Hong, Heejoon Myung

**Affiliations:** 1 Department of Bioscience and Biotechnology, Hankuk University of Foreign Studies, Gyung-Gi Do 17035, Korea; hyunento@hufs.ac.kr (H.K.O.); mango_virus@hufs.ac.kr (Y.J.H.); 2 LyseNTech Co. Ltd., Gyung-Gi Do 17035, Korea; hwhong@lysentech.com; 3 Bacteriophage Bank of Korea, Yong-In, Mo-Hyun, Gyung-Gi Do 17035, Korea

**Keywords:** *Enterococcus faecalis*, biofilm, phage, endolysin, antibiotic

## Abstract

*Enterococcus faecalis* is a Gram-positive pathogen which colonizes human intestinal surfaces, forming biofilms, and demonstrates a high resistance to many antibiotics. Especially, antibiotics are less effective for eradicating biofilms and better alternatives are needed. In this study, we have isolated and characterized a bacteriophage, PBEF129, infecting *E. faecalis*. PBEF129 infected a variety of strains of *E. faecalis*, including those exhibiting antibiotic resistance. Its genome is a linear double-stranded DNA, 144,230 base pairs in length. Its GC content is 35.9%. The closest genomic DNA sequence was found in *Enterococcus* phage vB_EfaM_Ef2.3, with a sequence identity of 99.06% over 95% query coverage. Furthermore, 75 open reading frames (ORFs) were functionally annotated and five tRNA-encoding genes were found. ORF 6 was annotated as a phage endolysin having an L-acetylmuramoyl-l-alanine amidase activity. We purified the enzyme as a recombinant protein and confirmed its enzymatic activity. The endolysin’s host range was observed to be wider than its parent phage PBEF129. When applied to bacterial biofilm on the surface of in vitro cultured human intestinal cells, it demonstrated a removal efficacy of the same degree as cefotaxime, but much lower than its parent bacteriophage.

## 1. Introduction

*Enterococcus faecalis* is a Gram-positive bacterium found in the intestinal tracts of mammals. *E. faecalis* is an opportunistic pathogen which has seen an increasing number of cases of antibiotic resistance being reported [1,2,3]. In immunocompromised patients, *Enterococcal* biofilm is frequently found in the gut and it is associated with hemorrhagic enteritis, food poisoning, and urinary tract infections [4,5,6]. The biofilm is also found in medical devices [7]. Bacteria inside a biofilm frequently secrete complex extracellular polymeric substances (EPSs), and interaction between these EPSs results in different characteristics of the bacteria than planktonic ones. Especially, antibiotics are not efficient for killing bacteria inside the biofilm [8]. Bacteriophages are viruses infecting bacteria which are useful as alternatives to antibiotics, especially for multidrug-resistant (MDR) pathogens. Many *Enterococcal* phages have been reported and used for the control of bacterial infections and biofilms [9,10,11,12]. 

Cefotaxime is a third-generation cephalosporin antibiotic active against Gram-positive and Gram-negative bacteria [13]. It is reported that the antibiotic showed a limited efficacy against *E. faecalis* [14], which makes it a good candidate for showing additive effects when used in combination with other modalities such as phages and endolysins.

Lytic phage has a gene encoding endolysin which is an essential component for phage burst from inside the host bacterium [15,16,17]. The enzyme’s role is to degrade the cell wall so that assembled phages can pass through them. In Gram-positive bacteria, the cell wall is the outermost component and purified endolysins can instantly kill the bacteria upon contact when added to the culture. Endolysins from *E. faecalis* phages have been previously purified and characterized a number of times [18,19,20,21]. Biochemical activity, as well as in vivo efficacy [18], was observed. In addition, some endolysins from phages of different hosts than *E. faecalis* were shown to be active against *E. faecalis* [22,23]. A synergistic effect of antibiotics and phage endolysin was also reported [24,25].

In this study, we compare the efficacy of the bacteriophage, its endolysin, and an antibiotic for the removal of *E. faecalis* biofilm on human intestinal cells cultured in vitro. 

## 2. Materials and Methods 

### 2.1. Bacterial Strains and Bacteriophage

The host bacterium used was *Enterococcus faecalis* CCARM5520 obtained from the Culture Collection of Antibiotic-Resistant Microorganisms in Korea [26]. Using this strain, phages were screened for infectivity from water samples obtained from the Opo waste water facility in Gwang-Ju, Gyung-Gi Do, Korea. Briefly, 4 ml of water sample was added to 100 μL of fresh cultured bacteria (OD^600^ = 0.5) and the mixture was grown using a soft agar overlay technique on an LB plate. Plaques were isolated after overnight incubation. One phage was selected, named PBEF129, and further characterized. For host range tests of the purified endolysin, nine *E. faecalis* strains, namely CCARM 5511, 5518, 5520, 5526, 5537, 5539, 5548, 5568, and 5569, from the Culture Collection of Antibiotic-Resistant Microorganisms in Korea and ATCC 19433 were used. The antibiotic resistance profiles of these strains are provided in Table 1. The *E. faecalis* strain expressing green fluorescence protein (*E. faecalis* OG1RF harboring pMV158GFP) was a generous gift from professor SS Yoon [27].

### 2.2. Bacteriophage Propagation and Purification 

Standard phage techniques were used as previously described [28]. Briefly, dilutions of phage suspension were mixed with host bacteria in a dilute, molten agar matrix (the “top agar” or “overlay”) containing 0.7% (*w*/*v*) agar in LB broth (Duchefa, Haarlem, Netherlands) which was distributed evenly to solidify on a standard agar plate (the “bottom agar” or “underlay”). After overnight incubation, plaques were visualized. The phages were purified using centrifugation and a glycerol gradient method, as described elsewhere [29]. 

### 2.3. Genomic Analysis of Bacteriophage PBEF129

Genomic DNA was isolated from purified phage PBEF129 particles (300 μL of 1 × 10^8^ PFU/mL) using the Phage DNA Isolation Kit (Norgen, Thorold, ON, Canada). Isolated genomic DNA was subjected to whole-genome sequencing using Illumina MiSeq (LAS, Gyung-Gi Do, Korea). The genomic library was prepared using the Truseq® Nano DNA Sample Preparation kit and sequenced to yield 150 bp paired-end DNA reads. Preprocessing to obtain trustworthy reads was performed by Skewer [30]. Then, the sequencing platform-derived errors were corrected by Karect [31] with the option -matchtype=hamming, which dealt with substitution errors only for the Illumina dataset. Viral genome assembly and annotation reconstruction of viral genomes were performed by SAVAGE [32], which performed viral quasispecies assembly using the overlap graph assembly paradigm. The assembled genome was remapped with the whole-genome sequence reads before conducting manual curation. Finally, annotation of protein coding genes was carried out using BLAST+ [33] against COG, SwissProt, and Pfam. Open reading frames (ORFs) were identified using PATRIC [34]. CLC genomics workbench 11 was used for phage DNA plotting. For domain searches of the phage endolysin, the Conserved Domain Search Service (CD Search) tool (https://www.ncbi.nlm.nih.gov/Structure/cdd/wrpsb.cgi, 15 July 2020) was used. A phylogenetic tree among related phages was drawn on the basis of the genes encoding phage tail fibers using Mega X (version 10.5). MAUVE was used to compare the genomic DNA from closely related phages.

### 2.4. Cloning and Purification of Recombinant Phage Endolysin

The gene encoding ORF 6, which was annotated as a phage endolysin, was PCR-amplified with the primers Forward: 5’ GCGGCGCATATGGCAGGAGAAGTATTTAGT 3’ and Reverse: 5’ GCGGCGCTCGAGAGATTTTTTAGTAATACC 3’. The PCR conditions were as follows: initial activation of pfu DNA polymerase at 94 °C for 2 min, followed by 30 cycles of denaturation at 94 °C for 20 s, annealing at 55 °C for 2 min, and extension at 72 °C for 5 min. The PCR product was cloned in an expression vector of pET21-a(+) using restriction enzyme sites NdeI and XhoI. The resulting recombinant plasmid was used for transformation into an expression strain, *Escherichia coli* BL21 (DE3)pLyss (Promega, Madison, Wisconsin, USA). Overexpressed N-terminal 6X histidine-tagged recombinant protein was purified using a Ni-NTA column (Qiagen, Hilden, Germany) according to the manufacturer’s instructions. The recombinant protein was eluted in 200 mM imidazole, pH 7.5, and the final eluate was dialyzed against 20 mM tris-Cl, pH 7.5.

### 2.5. Biochemical Activity (amidase) Test of Purified Endolysin

For biochemical enzymatic activity, since ORF 6 was annotated as an amidase, an amidase assay was performed by means previously indicated [35]. Briefly, purified endolysin was incubated in 50 mM sodium phosphate buffer, pH 7.2, containing 100 mM acetamide solution and 500 mM hydroxylamine solution at 37 °C for one hour. Then, a color reagent solution containing 6% (*w*/*v*) ferric chloride in 5.7% (*v*/*v*) hydrochloric acid was added, forming acethydroxamate, which was measured by colorimetric detection at 500 nm. Reagents were purchased from Sigma-Aldrich, USA. A standard curve was plotted before the actual amidase assay of the purified protein according to the protocol [35]. Endolysin activity was also visualized by using zymography. Freshly grown *E. faecalis* was added to a 15% acrylamide gel. Purified endolysin was loaded onto the gel and electrophoresis was performed in a non-denaturing condition at the maximal power of 130 V for 3 h and stained with Coomassie Brilliant Blue. The gel was washed using distilled water at room temperature for 20 min. A second wash was performed with 0.1% triton X-100 in PBS for 1 h. Finally, a third wash was performed with PBS.

### 2.6. Antibacterial Activity Test of Purified Endolysin on Live Bacterial Cells

Time and dose dependency of antibacterial activity were observed on live bacterial cells. The standard reaction buffer used was 20 mM Tris-Cl, pH 7.5. When freshly grown *E. faecalis* CCARM 5520 in a brain heart infusion (BHI) reached a cell density of 8.8 × 10^8^ CFU/mL as measured using hemocytometer, 1 ml cells were harvested using low-speed centrifugation (1000× *g* for 2 min) with the supernatant being discarded. The pellet was resuspended in the standard reaction buffer with the addition of purified endolysin and the mixture was incubated at 37 °C for six hours. The number of surviving cells was counted on a BHI plate. The minimum inhibitory concentration of the endolysin on *E. faecalis* CCARM 5520 was determined using the methods suggested by the Clinical and Laboratory Standards Institute (CLSI) [36].

### 2.7. Human Cell Line and Formation of a Bacterial Biofilm In Vitro

A human intestinal cell line, SNU-C4, was obtained from the Korean Cell Line Bank at Seoul National University, Korea. Briefly, 3 × 10^6^ cells were seeded in a 6-well plate containing RPMI 1640 (Fischer Scientific, Rockingham County, New Hampshire, USA) supplemented with 10% fetal bovine serum (FBS, Gibco, Dún Laoghaire, Dublin, Ireland), and the cells were allowed to grow at 37 °C in a CO_2_ incubator set at 5% CO_2_ until confluency was observed. An exponentially grown culture of *E. faecalis* expressing GFP—*E. faecalis* OG1RF harboring pMV158GFP (*EF*/green)—was harvested and resuspended in RPMI 1640 media, and 1 × 10^9^ CFU were added to each well containing confluently grown intestinal cells. The mixture was incubated for 2 h for the bacteria to adhere to the surface of the intestinal cells, and then the mixture was washed with phosphate-buffered saline to discard any unbound bacteria. Finally, RPMI 1640 and FBS were added to the mixture and it was further incubated for 24 h for the biofilm to grow. Then, phage PBEF129 (1 × 10^10^ PFU per well), its endolysin (4.8 µM), cefotaxime (32 μg/mL, which was the minimum inhibitory concentration for the strain), or various combinations were added to the culture to observe the efficacy of biofilm removal. The mixture was incubated for 2 h. Then, the supernatant of each well was discarded and the wells were washed with PBS to remove all planktonic cells, followed by treatment with 1% triton X-100 (Sigma-Aldrich, Saint Louis, MO, USA) for 1 min. Then, the remaining bacteria were viewed under a laser scanning confocal microscope (Zeiss, Oberkochen, Germany). They were also counted on a solid agar medium as colony-forming units after recovery from the mixture. F-actin of human intestinal cells was immunostained with Phalloidin-iFluor 594 reagent (Abcam, Cambridge, United Kingdom cat. #176757).

### 2.8. Statistical Analysis

All the data are presented as the mean ± standard deviation (SD) of 3 independent experiments. Statistical significance was determined using Student’s *t*-test or Tukey’s test using proc glm in SAS (SAS Institute), with *p*-values < 0.05 representing significance. 

## 3. Results

### 3.1. Genomic Analysis of Bacteriopahge PBEF129 Infecting E. feacalis

**Figure 1:**
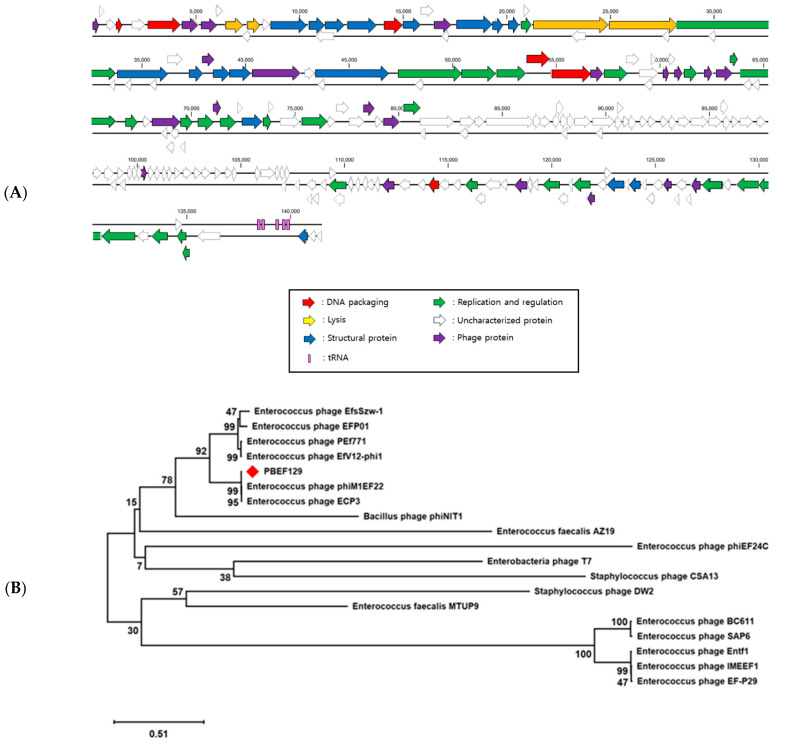

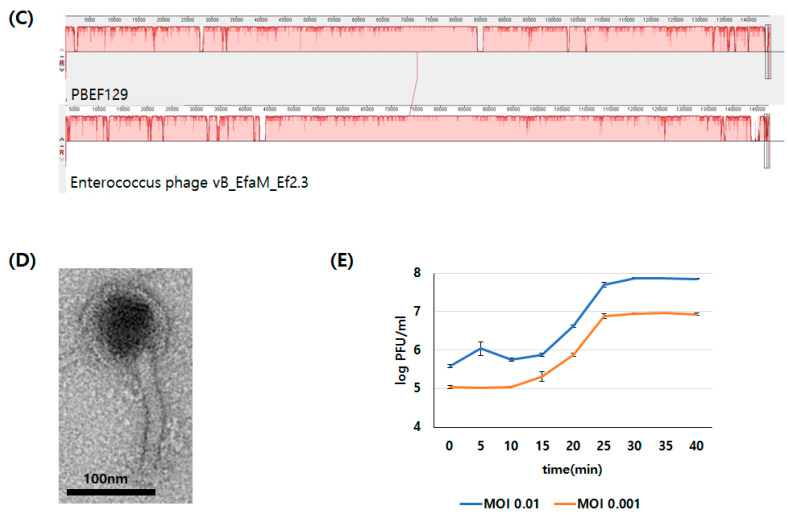
Genomic analysis of the bacteriophage PBEF129. (**A**) Open reading frame (ORF) map. Each arrow represents a functionally annotated ORF color coded according to its function. Blue, structural protein; green, replication and regulation; yellow, lysis; red, DNA packaging; violet, unspecified phage protein; white, uncharacterized. (**B**) Genomic tree showing distances among related phages. Red rhombus indicates PBEF129. The tree was drawn based on the genes encoding tail fibers of each phage. Bootstrap values are shown. (**C**) Alignment of whole genomes of phage PBEF129 and the closest phage vB_EfaM_Ef2 using MAUVE. (**D**) TEM image of phage PBEF129 shown with a scale bar. (**E**) One-step multiplication of phage PBEF129.

The genome of phage PBEF129 is a linear, double-stranded DNA, 144,230 base pairs in length (GenBank accession number MN854830.2). Its GC content is 35.9%. The closest genomic DNA sequence was found in *Enterococcus* phage vB_EfaM_Ef2.3, belonging to Herelleviridae, with a sequence identity of 99.06% over 95% query coverage from a BLASTN search. A total of 209 open reading frames (ORFs) were found and 75 were functionally annotated (Supplementary Table S1). Six were related to DNA packaging, four were related to lysis, 27 were related to replication and regulation, 17 were related to structural proteins, and 21 were annotated as phage proteins with unspecified functions. Five tRNA-encoding genes were also found. It was also verified using tRNAScan-SE [37]. The ORF map is shown in Figure 1A. Based on genes encoding tail fibers of related phages, a tree showing their relatedness was drawn (Figure 1B). Many *Enterococcus* phages were found to be closely related, but phages infecting *Bacillus, Enterobacteria*, or *Staphylococcus* were found to be distantly related. When comparing the genomes of phage PBEF129 and the closest phage vB_EfaM_Ef2.3 (Figure 1C), the arrangement of genes was linearly correlated, suggesting that the two phages were divergent due to the accumulation of point mutations over a long time rather than due to horizontal genetic exchange (mosaicism) [38]. Based on the morphology seen with the transmission electron microscope (TEM), phage PBEF129 belonged to the Siphovirus morphotype (Figure 1D). One-step multiplication of the phage showed a lytic cycle of 30 min and a burst size of 83 (Figure 1E).

### 3.2. Biochemical Characterization of Enzymatic Activity of Putative Endolysin from Phage PBEF129

**Figure 2:**
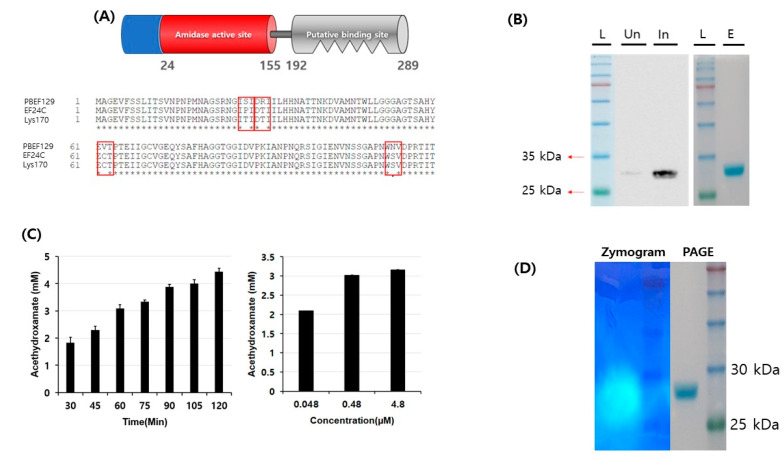
Purification and enzymatic activity of endolysin. (**A**) Suggested domain structure of the endolysin of phage PBEF129 from BLAST domain search. The total length of the endolysin is 289 amino acids. The putative amidase active domain (red) is from 24 to 155 amino acids and the putative peptidoglycan binding domain (gray) is 192 to 289 amino acids. Amino acids’ sequence alignment among related endolysins, PBEF129, EF24C and Lys170 [17], is seen under the domain diagram. Different amino acid residues in sequences are shown in the box. Only four amino acid residues differ in the enzymatically active domain (EAD). (**B**) Purification of the recombinant endolysin. Right panel, purification of putative endolysin. L, pre-stained protein ladder; E, eluted fraction. Left panel, Western blot analysis of the purified protein using anti-6XHis antibody. L, pre-stained protein ladder; Un, un-induced culture lysate; In, induced culture lysate. (**C**) Left: confirmation of amidase activity of the purified protein with various incubation times; 4.8 μM of protein was used for each time point. Right: confirmation of amidase activity of the purified protein with various amounts of protein. One-hour incubation was used for each enzyme concentration. (**D**) Left: zymogram of purified endolysin run on a non-denaturing PAGE containing the target bacteria. Right: PAGE analysis of the endolysin followed by Coomassie Brilliant Blue staining.

Of the 75 ORFs annotated, ORF 6 (GenBank accession no. QHJ73506.1) was annotated as an N-acetylmuramoyl-L-alanine amidase (endolysin) (Supplementary Table S1). The length of the protein is 289 amino acids and the closest related protein is the putative endolysin in *Enterococcus* phage vB_EfaM_Ef2.3 (GenBank accession no. MK721192.1), with a sequence identity of 99.31% over 100% query coverage from a BLAST search. An enzymatic active domain (EAD) was found at the N-terminus while a cell wall binding domain (CBD) was found at the C-terminus using BLAST domain searches (Figure 2A). When compared to two closely related endolysins, EF24C (GenBank accession no. BAF81277) and Lys170 (GenBank accession no. YP_001504118) [17], amino acid residues 27, 30, 62, and 113 were different in the amidase active domain. To determine its enzymatic activity, the gene encoding ORF 2 was PCR-amplified and cloned to an expression vector. A 6XHis-tagged form of the protein was overexpressed and purified to near-homogeneity using affinity chromatography (Figure 2B). Since the assay method for enzymatic activity of amidase is performed by measuring the amount of reaction product, acethydroxamate, using optical density at 500 nm, we first prepared a standard curve (Supplementary Figure S1). Then, we could observe amidase activity from the purified protein both in time- and concentration-dependent manners (Figure 2C). The amidase activity was also confirmed using zymography (Figure 2D).

### 3.3. Characterization of Antibacterial Activity on Live Bacterial Cells

**Figure 3:**
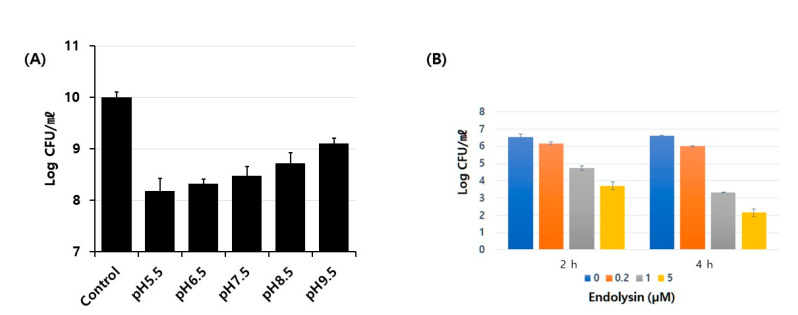
Characterization of antibacterial activity of the endolysin on live bacterial cells. (**A**) Optimal pH of the antibacterial activity of the endolysin. Buffer containing each pH was used for incubation of the antibacterial reaction with 4.8 μM of protein and 1-hour incubation. (**B**) Antibacterial efficacy was shown in a dose-dependent manner. Log reduction of bacterial count is shown with the amount of protein used indicated. Assay mixture at pH 7.5 was incubated at 37 °C for 2 or 4 hours.

We first checked what the optimal pH was for antibacterial activity of the endolysin. A range of pH values were tested from 5.5 to 9.5, and stronger activities were observed at lower pH (Figure 3A). To confirm the specificity of the antibacterial activity, we next performed a series of tests with different concentrations of endolysin for different periods of incubation time. As seen in Figure 3B, the antibacterial activity was shown to be both dose- and time-dependent, suggesting that the activity was specific for the target bacteria. Treatment of bacterial cells with the endolysin at the final concentration of 5 M for 4 h led to a 4 log reduction. The purified protein was active against an array of *E. faecalis* strains, including antibiotic-resistant strains, some of which the parent phage could not infect (Table 2). The minimum inhibitory concentration of the endolysin was >128 μg/mL, as measured using the standard protocol from the Clinical and Laboratory Standards Institute (CLSI) guidelines.

### 3.4. Comparison of Removal Efficiency Among Cefotaxime, Bacteriophage PBEF129, Its Endolysin, and their Combinations on E. faecalis Biofilm Formed on Human Intestinal Cells Grown In Vitro

**Figure 4:**
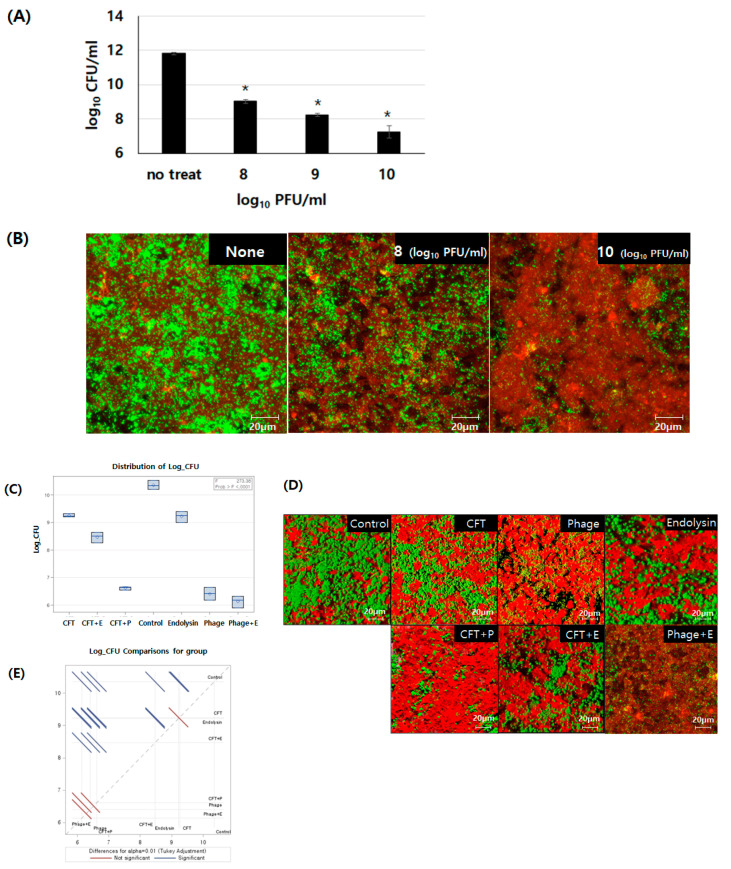
Biofilm removal efficacy of each antibacterial agent on in vitro cultured human intestinal cells. (**A**) After treatment with various concentrations of phages, the remaining bacterial load was recovered and counted as colony-forming units (CFUs). * *p* < 0.05. (**B**) Observation of remaining bacterial load after phage treatment on biofilm formed on top of in vitro cultured human intestinal cells. *E. faecalis* expressing green fluorescent protein (GFP) is shown in green, while human intestinal cells immunostained with anti-F actin antibody are shown in red. (**C**) After treatment with each agent, the remaining bacterial load was recovered and counted as colony-forming units (CFUs). CFT, cefotaxime; E, endolysin; P, phage PBEF129. Control is where the biofilm was treated with no agent. The experiment was performed in triplicate. (**D**) Observation of remaining bacterial load after each treatment on biofilm formed on top of in vitro cultured human intestinal cells. *E. faecalis* expressing green fluorescent protein (GFP) is shown as green, while human intestinal cells immunostained with anti-F actin antibody are shown as red. (**E**) Analysis of statistical significance among each treatment. The diffogram shows the result of Tukey’s test. Blue lines mean *p* < 0.01.

Intestinal colonization of *E. faecalis* is known to be associated with biofilm formation [39,40,41]. To eradicate the bacterial colonization, we need a modality which is effective for the removal of biofilms. Although the actual intestinal surface is covered with a mucin layer, chronic colonization of pathogenic bacteria would build a biofilm on top of the epithelial cells, leading to establishing an in vitro model of biofilm on top of cultured epithelial cells in this experiment. The efficacy of a less effective antibiotic, cefotaxime, was compared with alternatives to antibiotics, i.e., a bacteriophage, its endolysin, or their combination, in biofilm removal by counting residual bacteria after each treatment (Figure 4). First, we confirmed that phage treatment of a bacterial biofilm on an animal cell culture reduced bacterial load in a dose-dependent manner (Figure 4A,B). Treatment of the biofilm with cefotaxime or phage endolysin resulted in a limited reduction (~1 log) in bacterial load from the biofilm (Figure 4C). However, phage treatment was shown to be much more effective, with a 4 log reduction in bacteria. Combination treatment of cefotaxime and endolysin (CFT + E) showed a synergistic effect, while other combinations showed no synergistic effect. We also observed whether there was any morphological change in biofilm after each treatment under a confocal laser scanning fluorescence microscope (Figure 4D). On top of the confluently cultured human intestinal cells (red), *E. faecalis* (green) was seeded and incubated until a biofilm was formed. Then, each antibacterial agent or a combination of them was applied and removal of the biofilm was observed. Consistent with the results from the bacterial counting experiments, cefotaxime or phage endolysin removed bacteria to a limited extent, while application of phage PBEF129 to the biofilm resulted in a significant reduction in bacterial load.

## 4. Discussion

*E. faecalis* infection often leads to intestinal colonization, and it is involved in biofilm formation. In addition, multidrug-resistant pathogenic strains are frequently isolated in clinical settings. Vancomycin-resistant *Enterococci* (VRE) are considered one of the major threats in pathogenic microbiology. Thus, new modalities other than current antibiotics are urgently needed and bacteriophages are one such candidate. A newly isolated phage PBEF129 infects a series of drug-resistant *E. faecalis* strains. Although PBEF129 is closely related to a previously reported *Enterococcus* phage, vB_EfaM_Ef2.3 (Herelleviridae) [42], in this study, we focused on the characterization of a putative endolysin encoded by phage PBEF129. 

Usually, an endolysin belongs to one of four different classes of enzymatic activity in cell wall degradation: amidase, endopeptidase, lytic transglycosylase (lysozyme-like muramidase), or glucosaminidase [43,44]. From the amino acid sequence of the gene product of ORF 2, it was suggested that the candidate was an amidase which cleaved the chemical bond between N-acetyl muramic acid (NAM) and the first amino acid of the peptide chain. We experimentally confirmed its amidase activity using biochemical methods. Its antibacterial activity was confirmed in vitro against a panel of *E. faecalis* strains. It is not unusual that an endolysin shows a broader spectrum of target bacteria than its parent phage [22,44]. A phage’s host range is mainly determined by the presence of the receptor on the bacterial surface and myriad other factors which can also affect the host range. However, an endolysin only needs to recognize the cell wall before enzymatic degradation, so the presence of a specific receptor is not required. Accordingly, we also confirmed a broader host range for the endolysin than its parent phage, PBEF129. The fact that the endolysin was more effective against target bacteria at lower pH may reflect a favorable surface charge alteration of target bacteria for the endolysin. The anionic nature of teichoic acids and wall-associated proteins on cell walls would change as pH changes, possibly leading to a stronger ionic interaction between the cell wall and the endolysin. There are several reports demonstrating higher activities of endolysins at lower pH levels [45,46,47].

In terms of antibacterial killing efficacy, this endolysin showed a relatively weak activity compared to other *E. faecalis* phage endolysins reported previously. For example, the LysEF-P10 endolysin showed a 6.5 log reduction in bacterial count (CFU/mL) in an hour [16], while this endolysin showed a 3 log reduction in two hours for cultured bacterial cells. In addition, the MIC could not be obtained using this endolysin. 

Combination treatment using a lytic phage and an antibiotic was reported to show a synergistic effect [48]. Combination treatments of phage vB_AbaP_AGC01 and ciprofloxacin or gentamycin decreased *Acinetobacter* count 10 times more than separate treatments. This was not the case for phage PBEF129 in this study. Nonetheless, a combination treatment using the endolysin and an antibiotic showed a synergistic effect and it was demonstrated in a previous report [49]. An in vivo study using a *Pneumococcal* mouse model showed a higher survival rate (100%) with the combination of the endolysin Cpl-711 and cefotaxime than separate treatments (58%). A synergistic effect was also shown for the endolysin reported in this study, suggesting that the weak activity of an endolysin can be complemented by the addition of an appropriate antibiotic. 

There are several reports describing various phage endolysins’ efficacy against bacterial biofilms where only limited activities were reported [50,51,52,53]. One report described an endolysin against *Staphylococcus aureus* biofilm on the surface of a medical device which showed no apparent efficacy [54]. In a case where an endolysin and a polysaccharide depolymerase from a phage against *S. aureus* were used simultaneously, biofilm was effectively removed [55]. Antibiotics are usually considered much less effective at removing bacterial biofilm than bacteriophages [56,57]. Since many phages, if not all, have polysaccharide depolymerases in their virion, they have more chance to confront bacterial cells inside extracellular polymers surrounding bacteria [29]. Antibiotics or endolysins do not harbor depolymerase activity, resulting in poor efficacy against biofilms. We could expect additive effects of combination treatment using bacteriophages and endolysins over each treatment individually, provided that bacteriophages armed with stronger depolymerase initially destroy extracellular polymer in biofilms, and endolysin then destroys the bacterial cells more efficiently than an antibiotic. 

Endolysins are also reported to inhibit the formation of biofilms [50]. An endolysin with amidase activity against *Listeria monocytogenes* prevented biofilm formation when co-incubated with the bacteria for four days. Thus, endolysins could be applied to prevent biofilm formation on the surfaces of medical devices, e.g., catheters. Endolysins were also effective against tooth infections, which was another example of efficacy against biofilm [6,58]. Endolysins could effectively remove *E. faecalis* from human teeth or dentin slices.

Currently, four independent clinical trials of endolysins are underway (www.clinicaltrials.gov, 24 February 2021, identifiers NCT03163446, NCT01746654, NCT03089697, and NCT02840955), in which endolysins against *Staphylococcus aureus* are being tested from skin infections to bacteremia. Many other Gram (+) endolysins such as the one given in this paper and Gram (−) endolysins are being studied and may provide novel approaches for treating antibiotic-resistant bacteria.

## Figures and Tables

**Table 1:** Antibiotic resistance profiles of *E. faecalis* strains used in this study.

Antibiotic (μg/mL)	*Enterococcus faecalis* Strains								
	CCARM 5511 *	CCARM 5518	CCARM 5520	CCARM 5526	CCARM 5537	CCARM 5539	CCARM 5548	CCARM 5568	CCARM 5569
Ampicillin	4	4	4	4	4	4	4		
Ciprofloxacin	≥2	≤1	≥2	≥2	≤1	≥2	≥2	1	≥2
Erythromycin		≥4	≥4	≥4	≥4			≥4	
Gentamycin	≥500	≥500	≥500	≥500	≥500	≥500	≥500	≥500	≥500
Levofloxacin	≥4	2	≥4	≥4	≤1	≥4	≥4	≥2	≥4
Linezolid	≤2	≤2	≤2	≤2	≤2	≤2	≤2	≤1	≤1
Penicillin	≥8	8	≥8	8	8	≥8	≥8	8	≥8
Rifampin	≥2	≤1	≤1	≤1	≥2	≥2	≤1	≤1	≤1
Streptomycin	≥1000	≤1000	≤1000	≤1000	≥1000	≥1000	≤1000	≤1000	≤1000
Quinupristin- dalfopristin	≥2	≥2	≥2	≥2	≥2	≥2	≥2	≥2	≥2
Teicoplanin	≤4	≤4	≤4	≤4	≤4	≤4	≤4	≤1	≤1
Tetracycline	≥8	≥8	≥8	≥8	≥8	≥8	≥8	≥8	≥8
Vancomycin	2	1	2	2	2	2	2	2	2

* CCARM number is for strains listed in the Culture Collection of Antibiotic-Resistant Microorganisms in Korea [26].

**Table 2:** Lysis of *E. faecalis* strain by phage vs. endolysin.

*E. faecalis* Strain	Phage PBEF129	Endolysin
CCARM5511	−	+
CCARM5518	+	+
CCARM5520	+	+
CCARM5526	+	+
CCARM5537	−	+
CCARM5539	+	+
CCARM5548	+	+
CCARM5568	+	+
CCARM5569	+	+
CCARM5571	+	+
ATCC19433	+	+

On a lawn of each bacterium, 20 μL of phage (10^9^ PFU/mL) or purified endolysin (1 mg/mL) was spotted and the formation of clear zone was observed.

## Data Availability

The data presented in this study are available on request from the corresponding author.

## Supplementary Materials

The following are available online at https://www.mdpi.com/1999-4915/13/3/426/s1, Figure S1: Standard curve of amidase enzymatic assay, Table S1: Functionally annotated open reading frames (ORFs) of phage PBEF129.
