# Supplementary material for: Comparison of Enterococcus faecalis Biofilm Removal Efficiency among Bacteriophage PBEF129, Its Endolysin, and Cefotaxime
Source: Viruses. 2021 Mar 7;13(3):426. doi: 10.3390/v13030426 (PMC7999683; doi:10.3390/v13030426)
Supplement: Supplementary file 1 [file viruses-13-00426-s001.pdf]

Figure S1. The standard curve of biochemical amidase assay.

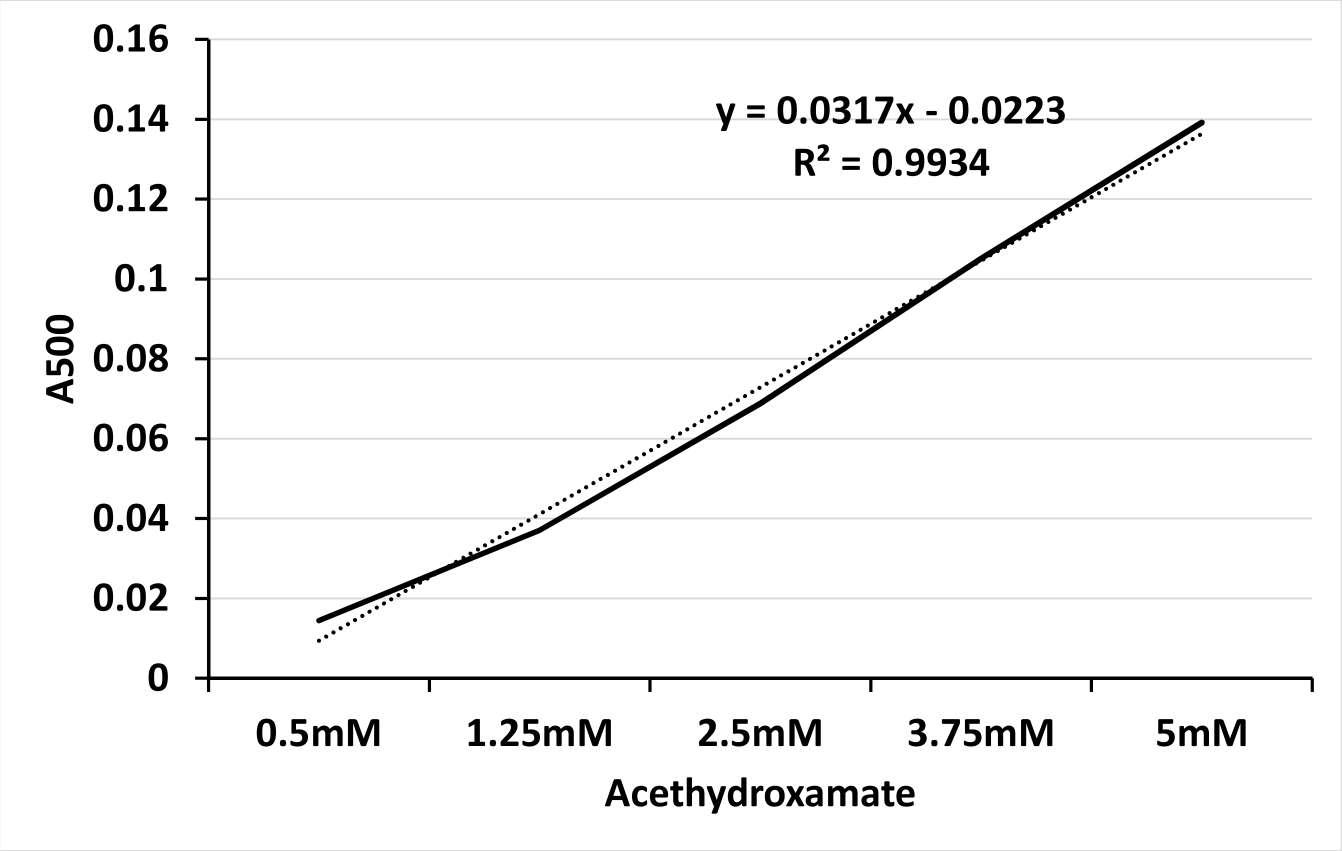

Table S1. Functionally annotated open reading frames (ORFs) of phage PBEF129

ORF	Function	Encoded phage protein	Related bacteria or phage	Query cover (%)	Identity (%)
8	Structural protein	putative portal protein	Enterococcus phage phiM1EF22	100	99.83
9		putative portal protein	Enterococcus phage phiM1EF22	100	99.83
10		major capsid protein	Enterococcus phage phiEF24C	99	99.57
11		baseplate hub assembly protein	Enterococcus phage PEf771	100	75.09
13		head completion protein	Enterococcus phage iF6	100	83.28
15		tail sheath protein	Enterococcus phage EF24C	99	99.47

**16**		tail tube protein	Enterococcus phage EFDG1	100	97.14
**17**		tail tape measure protein	Enterococcus phage EFDG1	100	72.61
**22**		capsid and scaffold protein	Enterococcus phage vB_EfaM_Ef2.3	99	99.63
**23**		tail tube terminator protein	Enterococcus phage iF6	100	78.21
**25**		putative baseplate	Enterococcus phage phiEF17H	100	99.57
**26**		putative baseplate J family structural protein	Enterococcus phage vB_EfaH_EF1TV	100	99.71
**28**		putative adsorption associated tail protein	Enterococcus phage phiM1EF22	100	99.91
**49**		flagellar M-ring protein	Enterococcus phage vB_EfaM_Ef2.1	100	99.69
**64**		tail length tape-measure protein	Enterococcus phage vB_EfaM_Ef2.3	100	67.59
**65**		putative adsorption protein	Enterococcus phage vB_EfaM_Ef2.1	100	99.12
**75**		structural protein	Enterococcus phage phiEF24C	100	99.11
**18**	**Replication & Regulation**	RNA polymerase	Enterococcus phage EF24C	100	99.48
**21**		putative glycerophosphoryl diester phosphodiesterase	Enterococcus phage vB_EfaM_Ef2.1	100	99.73
**29**		DNA helicase,phage-associated	Enterococcus phage vB_EfaH_149	100	99.80
**30**		putative transcriptional regulator	Enterococcus phage phiEF17H	100	99.82
**31**		helicase DnaB-like	Enterococcus phage EF24C	99	100.0
**35**		DNA primase	Enterococcus phage EF24C	99	99.7
**38**		putative resolvase	Enterococcus phage ECP3	100	99.55
**41**		putative integration host factor	Enterococcus phage EF24C	100	99.03
**42**		DNA polymerase I	Enterococcus phage EFLK1	100	99.88
**43**		DNA polymerase I	Enterococcus phage EF24C	100	99.43
**45**		Phage recombinase	Enterococcus phage 156	100	99.44
**46**		putative recombinase A	Enterococcus phage EF24C	100	100.00
**48**		putative sigma factor	Enterococcus phage ECP3	100	99.51
**50**		putative DNA polymerase	Enterococcus phage EFP01	100	68.71
**51**		metallophosphoesterase domain containing protein	Enterococcus phage vB_EfaM_A2	100	71.90

**54**		thioredoxin	Enterococcus phage vB_EfaM_Ef2.3	100	99.64
**56**		1-(5-phosphoribosyl)-5-amino-4-imidazole-carboxylatecarboxylase	Enterococcus phage phiM1EF22	100	99.58
**59**		putative GTP cyclohydrolaseII	Enterococcus phage vB_EfaH_EF1TV	100	98.84
**61**		putative serine/threonine protein phosphatase	Enterococcus phage phiEF17H	100	99.19
**62**		putative phosphoesterase	Enterococcus phage phiM1EF22	100	99.51
**68**		putative thymidylate synthase	Enterococcus phage phiM1EF22	100	99.68
**69**		putative anti-proliferative protein	Enterococcus phage ECP3	100	99.65
**70**		putative ribonucleotide reductase of classIb(aerobic),betasubunit	Enterococcus phage vB_EfaH_EF1TV	100	99.69
**71**		putative ribonucleotide reductase	Enterococcus phage ECP3	100	99.81
**72**		putative ribonucleotide reductase	Enterococcus phage EF24C	100	100.00
**73**		glutaredoxin-likeprotein	Enterococcus phage vB_EfaM_Ef2.3	100	98.75
**74**		repressor	Enterococcus phage vB_EfaM_Ef2.1	100	97.37
**6**	**Lysis**	N-acetylmuramoyl-L-alanine amidase	Enterococcus phage vB_EfaM_Ef2.1	100	99.31
**7**		peptidoglycan-bindingLysM	Enterococcus phage EF24C	100	99.53
**19**		putative tail lysin	Enterococcus phage phiM1EF22	99	100.00
**20**		putative tail lysin	Enterococcus phage EF24C	100	99.81
**2**	**DNA packaging**	terminase large subunit	Enterococcus phage EF24C	100	100
**3**		terminase large subunit	Enterococcus phage vB_EfaM_Ef2.1	99	99.61
**12**		prohead protease	Enterococcus phage EF24C	100	99.62
**32**		phage recombination exonuclease	Enterococcus phage vB_EfaH_149	100	99.72
**33**		putative exonuclease	Enterococcus phage phiEF17H	100	99.37
**58**		exonuclease	Enterococcus phage vB_EfaM_Ef2.1	100	87.78
**1**	**Phage protein (unspecified)**	phage protein	Enterococcus phage 156	100	98.59
**4**		phage protein	Enterococcus phage 156	100	97.71
**5**		phage protein	Enterococcus phage 156	100	99.58
**14**		phage protein	Enterococcus phage 163	100	70.34
**24**		phage protein	Enterococcus phage 163	100	80.00

**27**			phage protein	Enterococcus phage 156	100	80.00
**34**			phage protein	Enterococcus phage 156	100	99.80
**36**			phage protein	Enterococcus phage 156	100	99.10
**37**			phage protein	Enterococcus phage 156	96	98.04
**39**			phage protein	Enterococcus phage 156	100	96.86
**40**			phage protein	Enterococcus phage 156	100	99.24
**44**			phage protein	Enterococcus phage 156	100	99.53
**47**			phage protein	Enterococcus phage 156	100	98.44
**52**			phage protein	Enterococcus phage 156	100	98.81
**53**			phage protein	Enterococcus phage 156	100	99.30
**55**			phage protein	Enterococcus phage 156	100	97.53
**57**			phage protein	Enterococcus phage 149	100	92.98
**60**			phage protein	Enterococcus phage 156	100	97.39
**63**			phage protein	Enterococcus phage 163	98	54.39
**66**			phage protein	Enterococcus phage 156	100	98.62
**67**			phage protein	Enterococcus phage 156	100	96.50
**76**	**tRNA**	tRNA-Met-CAT				
**77**		tRNA-Pro-TGG				
**78**		tRNA-Arg-TCT				
**79**		tRNA-Trp-CCA				
**80**		tRNA-Asp-GTC				
